# Comment on “L-shaped association of selenium in blood metals mixtures with the incidence of liver fibrosis/cirrhosis: NHANES 2017-2020”

**DOI:** 10.1097/JS9.0000000000003396

**Published:** 2025-09-08

**Authors:** Chengting Zheng, Ping Chen, Kehang Dai

**Affiliations:** Gastrointestinal Department, Chongqing University Three Gorges Hospital, Chongqing, China

**HIGHLIGHTS**

- The correspondence raises concerns about the accuracy of vibration-controlled transient elastography (VCTE) in populations with obesity or other conditions affecting liver stiffness measurements, which may lead to misclassification of liver fibrosis or cirrhosis stages.
- We suggest that the authors address the limitations of VCTE, particularly in the context of the NHANES dataset’s diverse population with varying degrees of obesity and metabolic conditions.
- The correspondence recommends incorporating alternative diagnostic tools, such as magnetic resonance elastography or liver biopsy, to validate the findings and improve diagnostic accuracy.

*Dear Editor,*

We read with great interest the recent article titled “*L-shaped association of selenium in blood metals mixtures with the incidence of liver fibrosis/cirrhosis: NHANES 2017−2020*^[1]^.” The study provides valuable insights into the relationship between blood selenium levels and liver fibrosis/cirrhosis, highlighting the potential role of selenium as a protective biomarker for chronic liver diseases. We commend the authors for their comprehensive analysis using multiple statistical models to explore these associations. However, we would like to raise a methodological concern regarding the use of vibration-controlled transient elastography (VCTE) as a diagnostic tool for liver fibrosis and cirrhosis. This correspondence was developed following the TITAN Guidelines 2025^[2]^ for the declaration and use of artificial intelligence (AI) in scholarly publishing. No AI tools were employed in the conception, design, analysis, interpretation, or writing of this work.

While VCTE is widely employed for non-invasive liver stiffness measurement, its accuracy may be compromised in certain populations, particularly those with obesity or other conditions that affect liver stiffness measurements. Studies have shown that patients with higher body mass index or those with severe obesity may experience reduced accuracy with VCTE due to challenges in obtaining reliable measurements^[3]^. Additionally, factors such as liver congestion or hepatic inflammation may also affect the precision of VCTE results, potentially leading to misclassification of liver fibrosis or cirrhosis stages.

Given that the study population in the NHANES dataset includes individuals with varying degrees of obesity and metabolic conditions, we believe this issue warrants further consideration. Misclassification due to suboptimal VCTE performance could impact the study’s findings and limit the generalizability of the conclusions.

We suggest that the authors discuss this potential limitation more explicitly, acknowledging the possibility of misclassification and its implications for the study’s conclusions. Furthermore, the authors may consider incorporating alternative diagnostic methods, such as magnetic resonance elastography or liver biopsy, in future research to validate the findings from VCTE and improve diagnostic accuracy.

## Data Availability

The letter does not have any data.

## Research registration unique identifying number (UIN)

Not registered for the type of study.
